# Designing and Developing a Mobile App for Management and Treatment of Gestational Diabetes in Nepal: User-Centered Design Study

**DOI:** 10.2196/50823

**Published:** 2024-01-17

**Authors:** Aarthi Shanmugavel, Prabin Raj Shakya, Archana Shrestha, Jyoti Nepal, Abha Shrestha, Jean-Francois Daneault, Shristi Rawal

**Affiliations:** 1 Department of Health Informatics School of Health Professions Rutgers, The State University of New Jersey Piscataway, NJ United States; 2 Biomedical Knowledge Engineering Lab Department of Dentistry Seoul National University Seoul Democratic People's Republic of Korea; 3 Institute for Implementation Science and Health Kathmandu Nepal; 4 Department of Public Health Kathmandu University School of Medical Sciences Dhulikhel Nepal; 5 Department of Chronic Disease and Epidemiology Center of Methods for Implementation and Prevention Science Yale School of Public Health New Haven, CT United States; 6 Department of Obstetrics and Gynecology Dhulikhel Hospital Dhulikhel Nepal; 7 Department of Rehabilitation and Movement Sciences School of Health Professions Rutgers University Newark, NJ United States; 8 Department of Clinical and Preventive Nutrition Sciences School of Health Professions Rutgers, The State University of New Jersey Newark, NJ United States

**Keywords:** mHealth, mobile health, gestational diabetes, telehealth, usability testing, LMICs, low- and middle-income countries, user-centric design, social cognitive theory, South Asians, maternal health, diabetes, diabetes mellitus, daily glucose monitoring, hospital, medical institution, health center, clinical utility, Nepal, low income, clinical trial, focus group, interview, health care provider, medical practitioner, mobile app, application, digital health, app, apps, health education, web based, self-monitoring, glucose, physical activity, intervention

## Abstract

**Background:**

Mobile apps can aid with the management of gestational diabetes mellitus (GDM) by providing patient education, reinforcing regular blood glucose monitoring and diet/lifestyle modification, and facilitating clinical and social support.

**Objective:**

This study aimed to describe our process of designing and developing a culturally tailored app, *Garbhakalin Diabetes athawa Madhumeha*—Dhulikhel Hospital (GDM-DH), to support GDM management among Nepalese patients by applying a user-centered design approach.

**Methods:**

A multidisciplinary team of experts, as well as health care providers and patients in Dhulikhel Hospital (Dhulikhel, Nepal), contributed to the development of the GDM-DH app. After finalizing the app’s content and features, we created the app’s wireframe, which illustrated the app’s proposed interface, navigation sequences, and features and function. Feedback was solicited on the wireframe via key informant interviews with health care providers (n=5) and a focus group and in-depth interviews with patients with GDM (n=12). Incorporating their input, we built a minimum viable product, which was then user-tested with 18 patients with GDM and further refined to obtain the final version of the GDM-DH app.

**Results:**

Participants in the focus group and interviews unanimously concurred on the utility and relevance of the proposed mobile app for patients with GDM, offering additional insight into essential modifications and additions to the app’s features and content (eg, inclusion of example meal plans and exercise videos).The mean age of patients in the usability testing (n=18) was 28.8 (SD 3.3) years, with a mean gestational age of 27.2 (SD 3.0) weeks. The mean usability score across the 10 tasks was 3.50 (SD 0.55; maximum score=5 for “very easy”); task completion rates ranged from 55.6% (n=10) to 94.4% (n=17). Findings from the usability testing were reviewed to further optimize the GDM-DH app (eg, improving data visualization). Consistent with social cognitive theory, the final version of the GDM-DH app supports GDM self-management by providing health education and allowing patients to record and self-monitor blood glucose, blood pressure, carbohydrate intake, physical activity, and gestational weight gain. The app uses innovative features to minimize the self-monitoring burden, as well as automatic feedback and data visualization. The app also includes a social network “follow” feature to add friends and family and give them permission to view logged data and a progress summary. Health care providers can use the web-based admin portal of the GDM-DH app to enter/review glucose levels and other clinical measures, track patient progress, and guide treatment and counseling accordingly.

**Conclusions:**

To the best of our knowledge, this is the first mobile health platform for GDM developed for a low-income country and the first one containing a social support feature. A pilot clinical trial is currently underway to explore the clinical utility of the GDM-DH app.

## Introduction

Gestational diabetes mellitus (GDM), defined as hyperglycemia with onset during pregnancy, is a major public health issue worldwide. South Asians, who represent approximately one-fourth of the world’s population, are at a disproportionately higher risk of GDM [1-3], and the prevalence of GDM is increasing rapidly in South Asian countries, including Nepal [3-5]. Geographically situated between the two epicenters of the global diabetes epidemic, India and China [6], Nepal has a reported GDM prevalence ranging from 6.6% to 28% [7-9]. These estimates are alarming as GDM is associated with serious adverse perinatal outcomes and unfavorable long-term cardiometabolic consequences in both women and their children [10-14]. Although the short-term health and economic burden of GDM is substantial [15,16], its long-term implications are even more concerning, particularly among South Asian populations who are known to develop cardiometabolic complications at a relatively lower BMI than people with European ancestry [17-19]. Among women with GDM, those with South Asian ethnicity are also known to have a significantly higher risk of developing type 2 diabetes (T2D) compared to other ethnic groups [3,20]. Scalable and cost-effective solutions are thus needed to address the growing burden of GDM and its sequelae, particularly in low-resource South Asian countries, such as Nepal.

Successful GDM management relies on patient adherence to a complex care regimen, including dietary modification, adequate physical activity, weekly-to-biweekly antenatal follow-ups, and regular blood glucose monitoring and logging. Providing dietary and physical activity recommendations is a critical part of GDM management, but in resource-limited settings, such as Nepal, time for diet/lifestyle counseling often competes with other components of care. Face-to-face counseling for diet/lifestyle is also fraught with low participation rates and high attrition, as it is resource intensive for the health care providers and poses time and travel barriers for the patients [21,22]. Additionally, in many countries like Nepal, GDM counseling is only performed once, which is not conducive to facilitating a meaningful behavior change in diet and lifestyle. Mobile health (mHealth) technology provides new opportunities to circumvent these challenges [23] and support the treatment and management of GDM in low-resource settings. Mobile apps can aid in the management of GDM by providing patient education, reinforcing regular glucose monitoring and diet/lifestyle modification, and allowing health care providers to communicate and exchange health information with patients [24]. Mobile technology may offer cost-effective strategies to improve outcomes in patients with GDM by augmenting clinical care and empowering patients with GDM to self-manage their condition, yet this approach has not been tested previously in any low-income country [24].

App-based lifestyle interventions for GDM management are not common, especially in low-income countries, such as Nepal, where its prevalence is rapidly increasing [23,25,26]. To address this gap, our goal was to develop a mobile app that supports self-management and treatment among women with GDM in Nepal. In addition to making the app culturally tailored, our priority was also to design an app that matches the user needs and technological sophistication of the target users. Thus, taking a user-centered design approach [27], we developed the *Garbhakalin Diabetes athawa Madhumeha*—Dhulikhel Hospital (GDM-DH) app in collaboration with our target users, patients with GDM and providers, in Dhulikhel Hospital, a flagship university hospital in Dhulikhel, Nepal. Consistent with Bandura’s social cognitive theory (SCT) framework [28], the GDM-DH app supports GDM management by providing patient education, reinforcing regular blood glucose/carbohydrate monitoring, increasing self-efficacy for diet/lifestyle modification, and facilitating clinical and social support. Here, we aim to describe the app development process and features of the GDM-DH app.

## Methods

### Overall Study Design

**Figure 1:**
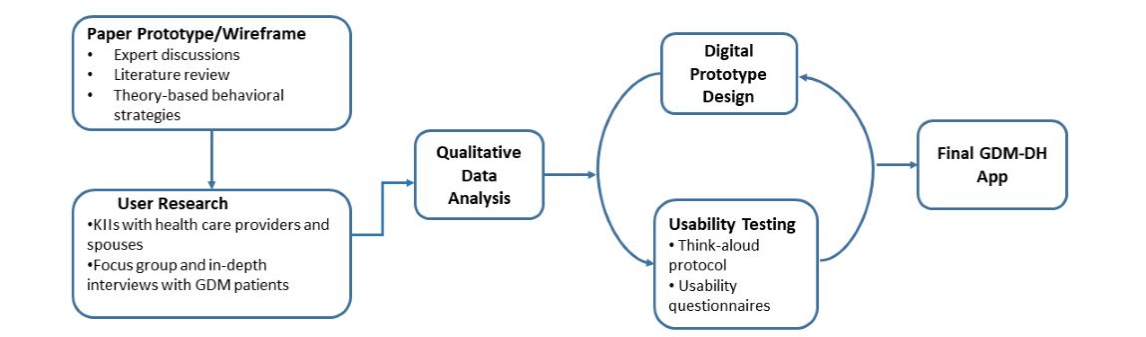
Schematic representation of the user-centered approach for GDM-DH app development among target users (women with gestational diabetes) in Dhulikhel Hospital, Nepal. GDM: gestational diabetes mellitus; GDM-DH: *Garbhakalin Diabetes athawa Madhumeha*—Dhulikhel Hospital; KII: key informant interview.

The study was conducted at Dhulikhel Hospital, a community-based tertiary-level university hospital of Kathmandu University (Nepal). We took a user-centered design approach to develop a culturally tailored mobile app (GDM-DH) for management of patients with GDM at the hospital. Figure 1 outlines the steps in GDM-DH app development. In the qualitative/requirement-gathering phase, patients with GDM were recruited for a focus group and structured interviews to show them the app prototype and obtain their feedback on its features and functions. Key informant interviews (KIIs) were conducted with clinicians and patients’ spouses. Incorporating and revising the app prototype based on user input, we built a minimum viable product (MVP), after which additional patients with GDM were recruited for usability testing including the think-aloud protocol [29]. The final GDM-DH app was developed following an iterative process of product design and user testing.

### Recruitment Procedure and Inclusion Criteria

Located in a periurban setting, about 20 km from the capital city of Kathmandu in Dhulikhel, Nepal, Dhulikhel Hospital has a catchment population of 1.9 million people and delivers approximately 3000 babies annually. All pregnant women receiving antenatal care at the Obstetric Outpatient Department at Dhulikhel Hospital undergo routine screening for GDM at 24-28 weeks of gestation. Inclusion criteria were pregnant women who (1) received antenatal care at Dhulikhel Hospital, (2) received a GDM diagnosis (within the preceding year), (3) owned a smart phone, and (4) could understand and read Nepali. Patients with a confirmed GDM diagnosis were recruited into the study with the help of a senior obstetrician-gynecologist (OB-GYN; coinvestigator in the study) and other staff in the OB-GYN department at Dhulikhel Hospital. A convenience sampling strategy was used to recruit participants meeting the aforementioned inclusion criteria for usability testing (n=18) and qualitative user research (n=19); for the latter, participants were recruited until data saturation was achieved.

### Ethical Considerations

The study protocol was approved by the Rutgers Newark Health Sciences Institutional Review Board (Pro2019001883) and the Ethical Review Board of the Nepal Research Health Council (NHRC; registration number 735/2019). Signed written informed consent was obtained from all participants by the research assistant at Dhulikhel Hospital. To ensure participant confidentiality, all documents including participant identifiers, such as the master list and consent forms, are stored separately in a locked cabinet and in a secure password-controlled Health Insurance Portability and Accountability Act (HIPAA)–compliant BOX folder. Only select research staff have access to the documents and folders containing participant identifiers and data. The participants (women with GDM and their spouses) received a mobile recharge card worth Nepalese rupees (NRs) 500 (US $3.77) to compensate for their time for the interview/focus group or usability testing.

### App Development Stages

#### Prototype Development

A multidisciplinary team including experts in GDM, mHealth, and behavior and implementation sciences, as well as health care providers and patients at Dhulikhel Hospital, contributed to the development of the GDM-DH app. Content modules and features to be included in the app prototype were selected based on a literature review, theory-based behavioral strategies, discussions with subject matter experts, and international recommendations and guidelines (including the Package of Essential Noncommunicable [PEN] disease interventions for primary health care in low-resource settings) [30]. A series of meetings and a full-day workshop were conducted with the research team to select and finalize the features of the app prototype. During the meetings, app features were selected based on expected user needs, alignment with theory-based constructs for behavior change, and the logistical and economic feasibility of incorporating these features in the app. The meetings and deliberations spanned over several weeks until differences were resolved and consensus was reached.

#### Qualitative User Research

After finalizing the app’s content and features, a focus group and structured interviews were conducted to explore the perceived barriers to and facilitators of GDM management and to seek feedback on the GDM app prototype. A total of 12 women with a GDM diagnosis (either current or in the preceding 1 year) were recruited from Dhulikhel Hospital, 4 (33.3%) of whom participated in a focus group and the remaining 8 (66.7%) in structured interviews. KIIs were also conducted with health care providers (n=5) and spouses of patients with GDM (n=2). All interviews were audio-taped and transcribed verbatim. A thematic analysis [31] of the interviews was performed to generate interview themes and memos.

The focus group and interviews explored the in-depth understanding of the target users’ views and opinions about GDM and its management, including knowledge and treatment gaps, perceived self-efficacy and barriers to GDM management, strategies to increase adherence to dietary/lifestyle management of GDM, and related social, cultural, and environmental factors. At the end of the focus group/interviews, the participants were given a demonstration of the app’s wireframe prototype, which is a schematic illustration that shows the app’s proposed interface, navigation sequences, and features and function. Feedback was then solicited from patients/providers/spouses on (1) the app dashboard, layout, and navigation; (2) usefulness of app features; (3) data entry burden; (4) usefulness of educational modules covered (5) clarity of graphs and data visualizations; and (6) additional features and content.

#### Usability Testing

Incorporating and revising the app prototype based on user input, we built an MVP, the simplest-possible version of the GDM-DH app, which retained the key features and functionalities of the app. The MVP was user-tested with 18 patients with GDM via the think-aloud protocol [29]. Individual 1-on-1 usability testing sessions were conducted in a private space and overseen by 2 facilitators; 1 facilitator led the session, while a designated notetaker recorded patients’ verbalizations. Usability testing consisted of a 2-step think-aloud protocol [32], in which the patients were asked to verbalize their thoughts as they navigated and completed various specified tasks (eg, profile setup, diet entry, weight visualization review, open video lesson) on the app. Patients were also asked to rate the difficulty of completing each task on a 5-point scale ranging from very easy to difficult. Additionally, they were asked to provide feedback on how the features and functions of the app could be improved upon [33]. Content analysis [34] was used to analyze and summarize the notes and verbalizations from the think-aloud protocol.

#### Final GDM-DH App Development

The facilitators’ notes and observations from the usability testing were compiled and scanned for indicators of usability problems experienced by the patients, such as annoyance, doubt, confusion, and slow/incomplete task completion. Based on the findings of the usability testing and recommendations provided by patients with GDM, a list of recommended modifications was compiled and discussed with the app developer (Ayata Inc, Kathmandu, Nepal). The results were used by the app developer to address the key usability barriers and patients’ preferences/feedback and develop a final version of the GDM-DH app for testing in a pilot clinical trial.

## Results

### Prototype and Content Development

Based on discussions with subject matter experts over a series of conference calls and workshops, we decided that the GDM-DH app suite would have 2 components: a mobile app for patients and a web-based portal for health care providers and researchers. The features and functionalities of the GDM-DH app were guided by Bandura’s SCT [28], which was selected as it has been widely applied in the dietary/lifestyle management of chronic health conditions [35] and is shown to be a suitable framework for promoting healthy behaviors among pregnant women, including those with GDM [36,37]. We focused our intervention modules on the SCT constructs of *self-efficacy* (confidence in one’s ability to take action and overcome perceived barriers to a behavior change), *self-regulation/self-control* (ability to understand and manage feelings, behaviors, and actions to achieve goals), *behavioral capabilities* (knowledge and skills needed to perform a given behavior), *reinforcements* (responses to a person’s behavior that increase or decrease the likelihood of occurrences), and *outcome expectations/expectancies* (anticipated outcomes of a behavior and values a person places on the probable outcomes of a behavior) [38]. Behavior change techniques (BCTs) [39] for the GDM-DH intervention content were selected based on the published literature [40] that maps the BCTs with the SCT constructs for behavior change (eg, the BCT of information about health consequences aligns with the SCT construct of outcome expectations). The SCT constructs *observational learning* (acquiring a new behavior by watching someone else performing it and observing their outcomes) and *environment* (physically external factors that can influence a behavior) were not targeted, as it was not feasible to achieve them at this time using the mobile app.

Using the SCT framework for behavior change, we decided that the content and features included in the GDM-DH app would support self-management of GDM by (1) providing health education, (2) helping patients identify and set target health goals (for diet, physical activity, and glucose levels), (3) enhancing their self-efficacy to meet target goals, and (4) facilitating desired support from family members. In SCT, self-monitoring of behavior is the first and most important step in self-regulating appropriate behavior changes [28]. Self-monitoring is also known to be a powerful behavior change strategy for changing diet and physical activity [41]. Hence, we decided that the core features of the GDM-DH app would facilitate the users to record and self-monitor their blood glucose, blood pressure, carbohydrate intake, physical activity, and gestational weight gain (GWG). The app would use automatic feedback and data visualization to aid in self-monitoring, as well as innovative technological features to minimize the self-monitoring burden, including visual aids for estimating carbohydrate portion sizes and smartphone GPS and accelerometer sensors for obtaining physical activity data [42]. Health care providers would be able to use the web-based portal of the GDM-DH app to enter/review blood glucose readings, track patient progress, and accordingly guide treatment and counseling [42].

In many South Asian countries, women are not the sole health decision makers, with mothers-in-law and husbands having a strong influence on their health decisions during pregnancy [43-45]. Additionally, family members are closely involved in a pregnant woman’s food selection and preparation, thus influencing her dietary behaviors. Hence, the team decided that the GDM-DH app would be designed to garner social support from family members by allowing the patient to add friends and family members to the app, providing them with access to track and follow the patient’s progress toward stated goals.

### Educational Modules

The educational content for the app was adapted from international recommendations and guidelines, including PEN‎ disease interventions for primary health care in low-resource settings [30]. The topics covered information included on GDM and associated risk factors, short- and long-term health consequences of GDM for mother and child, clinical and lifestyle management of GDM, dietary and physical activity recommendations for GDM, the role of social support in GDM management, and the proper use of insulin and a glucometer. The educational materials for the app included text- and image-based materials written at less than an eighth-grade reading level and brief 5- to 10-minute videos narrated by the health care providers in Dhulikhel Hospital.

### Qualitative User Research

Qualitative findings from the focus group and interviews, which have been described in detail previously [46], provided insight into the app content and features, as well as design elements that needed to be added or modified. Briefly, we identified several facilitators of GDM management, including at the individual level (eg, concern for the baby’s health), family level (eg, spousal accompaniment to hospital visits, emotional support), and health system level (eg, universal GDM screening, team approach to management). Notable barriers included inadequate time for diet/lifestyle counseling during hospital visits, an abrupt change in the diet/lifestyle from pre- to post-GDM diagnosis, misconceptions around diet and physical activity, and social/cultural barriers, including food-centered traditions and festivities and a lack of decision-making power in the household. The majority of patients with GDM and their spouses indicated that they lacked sufficient information to manage GDM and were frustrated by frequent hospital visits.

They gave me a diet chart where it was written what to eat in the morning, daytime, and evening, but I didn’t receive any kind of other documents to read about my disease.

Patient with GDM on lack of information about GDM

They think that like hypertension or thyroid medication, if they start to take an insulin injection, they have to use it throughout their life, and they think it is required for serious conditions only…you know there are a lot of myths.

Provider on common misconceptions

There are traditions…you have to bless her and give her dai chiura; she has to eat many things. In that time, I think she ate curd and more sweet food; that is why earlier I think when I saw her last blood report, it was under control, but now again, it is all high. I think it is because she ate more during that time…these festivals…earlier too, her sisters came to bless, and she ate more curd and sweets, and again, she started eating rice instead of roti. So, I think that is why her blood sugar level is high again.

Spouse of a patient with GDM on social/cultural barriers to blood glucose management

All participants agreed that the proposed mobile app and features would be useful and relevant to women with GDM. They believed it would help overcome existing barriers by empowering pregnant women with information and tools to manage GDM and track their progress.

If we had seen this app before, I think we would have been able to control blood sugar levels, and we would have been able to plan. I think after seeing this, it would have helped, it would be useful.

Spouse of a patient with GDM on the GDM-DH mobile app

Just knowing that this app on its own records and tells you about your physical activity makes us alert…we will know how much more activity we need to do…it makes it easier.

Patient with GDM on the GDM-DH mobile app

However, both patients with GDM and health professionals requested more content with respect to medical management and diet/lifestyle modification for GDM. Based on findings from the qualitative study, we changed some app features and design elements (eg, data visualization), in addition to modifying the educational materials and other resources to further tailor the GDM-DH app culturally. For example, the educational modules were revised to address specific cultural and social challenges faced by our patients (eg, food-centered festivals, long-held dietary/cultural practices surrounding pregnancy), and appropriate strategies were provided to problem-solve around these barriers. Based on our target users’ suggestions, we added example meal plans with locally available and culturally staple foods, video demonstrations of safe and culturally relevant exercises during pregnancy (eg, yoga, mild hiking, walking), and revised visual aids for carbohydrate estimation to include standardized pictures of staple Nepalese foods with common portion sizes shown in locally used utensils, such as plates, bowls, and cups.

### Usability Testing

In total, 18 newly diagnosed patients with GDM participated in the usability testing [29] with the MVP. The mean age of patients in the usability testing was 28.8 (SD 3.3) years. All patients were married, and slightly more than half (n=10, 55.6%) were homemakers. The mean gestational age was 27.2 (SD 3.0) weeks, and the average number of years of schooling was 13.3 (SD 2.8) years.

Results from the think-aloud protocol are described in Table 1. The mean usability score across the 10 tasks was 3.50 (SD 0.55; maximum score=5 for very easy). The task completion rates ranged from 55.6% (n=10) to 94.4% (n=17) across the 10 tasks, with the lowest completion rate for the task requiring the patients to look up their next scheduled appointment on the app. All patients except 1 (5.6%) were able to successfully complete tasks requiring them to enter their weight and systolic and diastolic pressure into the app.

**Table 1:** Usability testing of key important features of the GDM-DH^a^ app among target users (women with GDM^b^) in Dhulikhel Hospital, Nepal (n=18).

Usability testing task	Successful completion, n (%)		Very difficult, n (%)	Difficult, n (%)	Normal, n (%)	Easy, n (%)	Very easy, n (%)	Score^c^, mean (SD)
	Yes	No						
Enter fasting blood glucose levels.	16 (88.9)	2 (11.1)	0	0	5 (27.8)	11 (61.1)	2 (11.1)	3.8 (0.6)
Enter postprandial blood glucose levels and view the glucose chart.	16 (88.9)	2 (11.1)	0	0	5 (27.8)	13 (71.2)	0	3.7 (0.5)
Enter the systolic blood pressure level.	17 (94.4)	1 (5.6)	0	0	6 (33.3)	10 (55.6)	2 (11.1)	3.8 (0.6)
Enter the diastolic blood pressure level and view the blood pressure chart.	17 (94.4)	1 (5.6)	0	0	5 (27.8)	13 (71.2)	0	3.7 (0.5)
Open a video on GDM nutrition.	16 (88.9)	2 (11.1)	0	2 (11.1)	3 (16.7)	12 (66.7)	1 (5.6)	3.7 (0.8)
Add a friend or family member in the app.	12 (66.7)	6 (33.3)	1 (5.6)	4 (22.2)	5 (27.8)	7 (38.9)	1 (5.6)	3.2 (1.0)
Enter weight.	17 (94.4)	1 (5.6)	0	0	4 (22.2)	12 (66.7)	2 (11.1)	3.9 (0.6)
Find out the daily step count from today.	12 (66.7)	6 (33.3)	1 (5.6)	3 (16.7)	6 (33.3)	7 (38.9)	1 (5.6)	3.2 (1.0)
Figure out how many carbohydrates were consumed at breakfast today.	12 (66.7)	6 (33.3)	0	4 (22.2)	6 (33.3)	8 (44.4)	0	3.22 (0.8)
Find out when the next appointment is.	10 (55.6)	8 (44.4)	2 (11.1)	5 (27.8)	5 (27.8)	6 (33.3)	0	2.8 (1.0)

^a^GDM-DH: *Garbhakalin Diabetes athawa Madhumeha*—Dhulikhel Hospital.

^b^GDM: gestational diabetes mellitus.

^c^Patients were asked to rate the difficulty of completing each task on a 5-point scale ranging from 5 for “very easy” to 1 for “very difficult.” The mean (SD) score represents an average score for the corresponding task across all patients.

### Modifications to the GDM-DH App After Usability Testing

Findings from the focus group, interviews, and usability testing were reviewed to identify recurring themes of feedback with respect to the GDM-DH app’s content, usability, navigation, and functionalities. These findings allowed us to gain insight into participants’ thought processes with regard to app use, places where they encounter difficulties, and ways to improve the app’s usability. As shown in Textbox 1, these themes served as valuable insights that were used by the app developer to address the key usability barriers and participants’ preferences/feedback and develop a final version of the GDM-DH app for testing in a pilot clinical trial. For example, considering that nearly half of the usability testing participants struggled in identifying their upcoming antenatal appointments, we decided that the upcoming appointments would be shown in a list, in addition to the calendar. Additionally, we included a comprehensive video tutorial on how to navigate the GDM-DH app and use its features. In the web-based portal, in addition to the features decided on by the research team and software development company, we incorporated new features requested by the providers, including a patient finder tool, data export customization, and additional analytics in the dashboard, such as the average number of antenatal visits per patient and the percentage of patients under medical therapy for GDM.

**Modifications requested and incorporated in the *Garbhakalin Diabetes athawa Madhumeha*—Dhulikhel Hospital (GDM-DH) app based on user research and usability testing among target users (women with gestational diabetes mellitus [GDM]) in Dhulikhel Hospital, Nepal.**

**Educational resources**

- Use simple and clear language in educational resources, avoiding jargon and using terms understandable to the target users.
- Provide clear guidance on healthy food selections and appropriate portion sizes, including example meal plans with locally available foods.
- Clarify common misconceptions around diet and physical activity during pregnancy.
- Address specific cultural and social challenges faced by target users with respect to diet/lifestyle modification and provide appropriate strategies to problem-solve around those barriers.
- Add information about the signs and symptoms of hypoglycemia, along with practical strategies for effective management.
- Clarify criteria for when insulin or medication is indicated for GDM.
- Add examples of physical activity and exercise videos that are appropriate for pregnant women.
- Include information about contraindications for physical activity during pregnancy, including warning signs to stop exercising.
- Add an educational module on how family members can provide support to women with GDM.

**App interface and features**

- Use bigger font sizes; enlarge and make the images clearer.
- Revise the visual aids to include standardized pictures of common food portion sizes in locally used utensils, such as plates, bowls, and cups.
- Add reminders to input blood glucose, weight, diet, and blood pressure data.
- Use pop-ups to confirm data input and avoid double entries.
- Use bar graphs instead of line graphs to present blood glucose, weight, and blood pressure visualizations.
- Make glucose, weight, diet, and blood pressure data visible both as a list and in graphical format.
- Show upcoming appointments in a list, in addition to the calendar.
- Add the hospital hotline number under Help and Support.
- Include a video tutorial on how to navigate the GDM-DH app and use its features.

**Web-based portal**

- Include a patient finder feature to search for a patient quickly.
- Add a function to customize data export based on specific parameters and layout.
- Add dashboard analytics based on patient data (eg, average number of antenatal visits per patient, percentage of patients under medical therapy for GDM)

The decision to retain or disregard the requested modifications was based on their potential influence on behavioral and clinical outcomes, budget feasibility, the implementation time frame, and their potential impact on the app’s scalability in the future. For example, meal plans were added because they could be easily incorporated with minimal time and cost but would have maximal health gains. However, our target users also suggested that we add a feature to connect and chat with other app users with GDM, as well as a platform to communicate directly with health care providers via the app; these features were not added due to logistical, technical, and funding constraints but may be considered in the future.

### Final GDM-DH App

Based on the SCT framework, the final version of our culturally tailored GDM-DH app supports GDM management by providing patient education, reinforcing regular blood glucose/carbohydrate/weight monitoring, increasing self-efficacy for diet/lifestyle modification, and facilitating clinical and social support. A detailed description and justification of the app features as well as their alignment with the SCT constructs are provided in Table 2.

**Table 2:** Description and justification of the GDM-DH^a^ app features designed for self-management and treatment of Nepalese women with GDM^b^.

Feature and description		Rationale		SCT^c^ constructs
**Educational modules**				
	Educational modules consist of text- and image-based materials and brief videos covering various health and nutrition topics related to GDM and its management.	The level of knowledge about GDM is significantly associated with self-management efficacy and glycemic control [47-49]. It also facilitates better information retention as patients can go through the lessons at their own pace and revisit them at their convenience.		Self-efficacy Behavior capabilities Outcome expectancies
**Blood glucose monitoring**				
	Blood glucose levels can be logged in for fasting and postprandial levels 3 times daily. These data are displayed in color-coded graphs (red and green bars for above- and within-target ranges, respectively).	Self-monitoring of glucose levels is associated with an increase in self-efficacy and better glycemic control [50]. Data visualizations increases patient awareness and helps health care providers with timely and informed clinical decision-making.		Self-regulation Self-efficacy
**Carbohydrate monitoring**				
	The app incorporates standardized pictures of local Nepalese foods with common portion sizes to help the user estimate and track calories and carbohydrate (grams) in their meals.	It builds the user’s self-efficacy for understanding and changing their carbohydrate intake patterns.		Self-regulation Behavior capabilities
**Blood pressure monitoring**				
	Users can log/track their systolic and diastolic blood pressure. These data are displayed in color-coded graphs (red and green bars for above- and within-target ranges, respectively).	Data visualization increases patient awareness and helps health care providers with timely and informed clinical decision-making.		Self-regulation Self-efficacy
**GWG^d^**				
	Based on weights entered by the user, the app creates a graph comparing the user’s weekly GWG rate with the recommended guidelines for optimal GWG, depending on the pre-pregnancy BMI.		Weight monitoring builds the user’s self-efficacy for understanding and managing their GWG.	Self-regulation Self-efficacy
**Physical activity**				
	The app integrates with the Google-Fit app to pull and graph physical activity data, including the step count.		It builds the user’s self-efficacy for understanding and changing their physical activity patterns.	Self-regulation Self-efficacy
**Appointment reminder**				
	The app has an in-built calendar, which the users can use to record and view upcoming antenatal appointments.	The reminder system enables patient adherence to the antenatal care regimen [51].		Reinforcement
**Social network**				
	Via a social network “follow” feature, the patient is able to list 1 or more friends/family members as contacts in the app and give them the permission to view their logged data or progress summary.	The app helps a user garner social support from friends/family and offers a source of accountability, motivation, and shared experience.		Reinforcement
**Web** **-based** **portal**				
	Health care providers can use the web-based admin portal to register a new patient, as well as enter, update, or review clinical and other patient-related information (glucose/blood pressure/weight/diet, clinical history/notes).	It streamlines the providers’ workflow, as they can quickly look at patient data visualizations to understand patient behaviors and progress and accordingly guide their treatment and counseling.		Reinforcement

^a^GDM-DH: *Garbhakalin Diabetes athawa Madhumeha*—Dhulikhel Hospital.

^b^GDM: gestational diabetes mellitus.

^c^SCT: social cognitive theory.

^d^GWG: gestational weight gain.

### Mobile App

**Figure 2:**
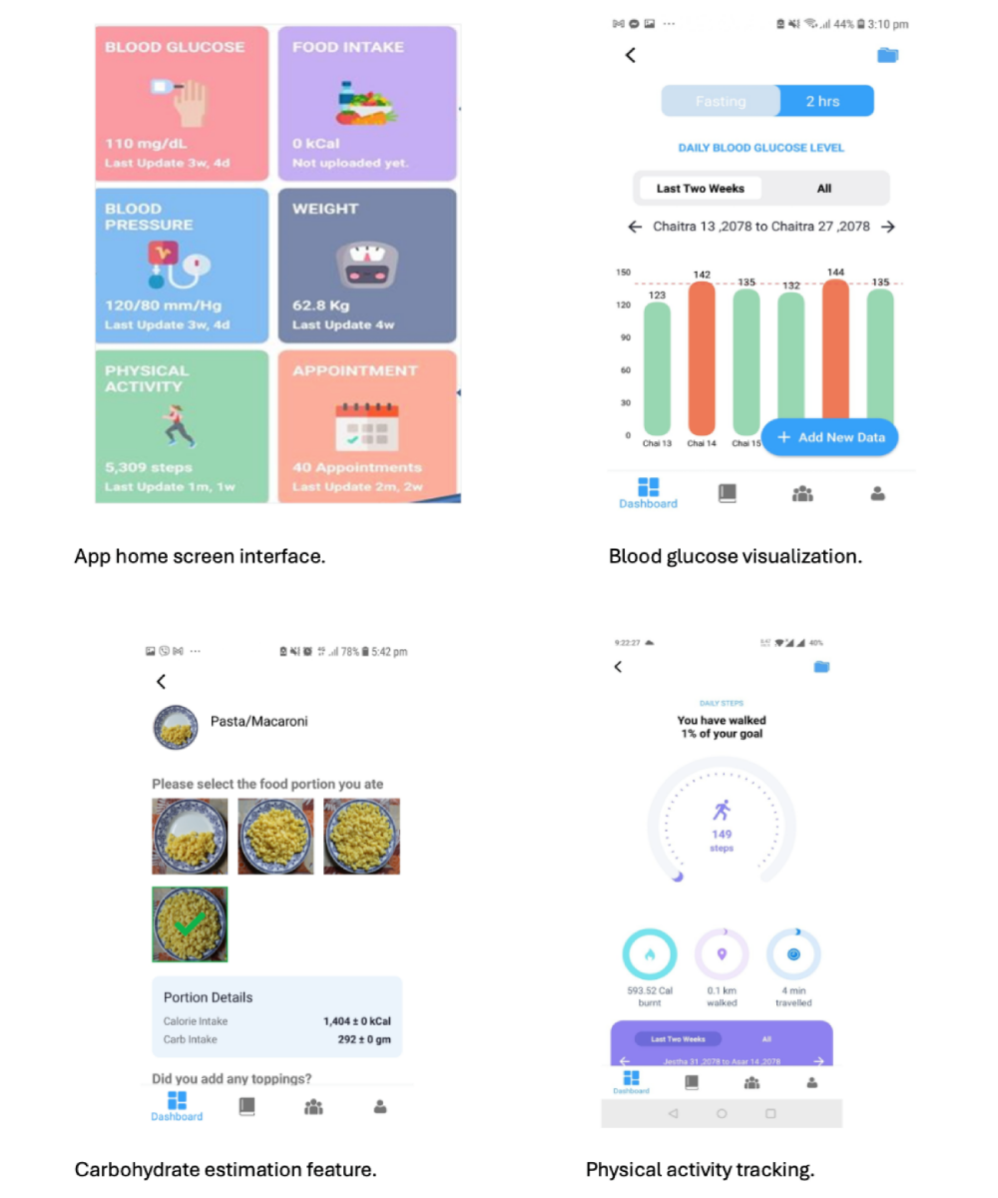
Key app features of the GDM-DH app designed for self-management and treatment of Nepalese women with GDM. GDM: gestational diabetes mellitus; GDM-DH: *Garbhakalin Diabetes athawa Madhumeha*—Dhulikhel Hospital.

The mobile app, which is patient facing, includes 6 feature icons on its home page: (1) Blood Glucose, (2) Food Intake, (3) Blood Pressure, (4) Weight, (5) Physical activity, and (6) Appointment (Figure 2). Using these features, the app allows patients to record and self-monitor their blood glucose, blood pressure, carbohydrate intake, physical activity, and GWG and track them over time. The app has a goal-setting feature and uses innovative technological features to minimize the self-monitoring burden, such as visual aids for carbohydrate estimation and integration with the Google-Fit app to automatically log physical activity data. Based on the data entered, the app provides automatic feedback about blood glucose, blood pressure, and GWG via a feedback engine that compares the user data to existing guidelines and recommendations. The app also generates visual displays summarizing their blood glucose, blood pressure, diet, physical activity, and weight patterns, allowing the user to easily monitor their alignment and progress toward target goals. In addition to the self-monitoring features, multimedia video- and text-based modules are included in the app as educational resources. The GDM-DH app also includes a social network “follow” feature, allowing the user to list 1 or more friends/family members as contacts in the app and give them the permission to view their logged data or progress summary. The app has an in-built calendar, which the users can use to record and view upcoming antenatal appointments.

### Web-Based Portal

The web-based portal can be securely accessed by researchers and health care providers from any device that supports modern web browsers. The web-based portal has features for patient management, data capture and review, and data dashboard/visualization. Health care providers can register a new patient; enter, update, or review clinical and other patient-related information (patient vitals, measurements, clinical notes, medications, etc); and schedule or make changes in appointments. Using Apache Kafka, the web-based portal syncs with the mobile app and allows providers to access data and graphs summarizing the patient’s diet, physical activity, weight, blood pressure, and blood glucose patterns. This streamlines the providers’ workflow and allows them to easily track patient progress and accordingly guide their treatment and counseling. Researchers and admin users can use the web-based portal to add new users, add/update the app modules/images/visualization, and audit changes made by users.

### Data Security

Data from the mobile app are stored in a HIPAA-compliant, secure server hosted by Amazon Web Services. MongoDB is used to implement the database service, which is a free, open source, no–Structured Query Language (no-SQL) database program tailored to support big data. It is encrypted and access-controlled using tokens to ensure it cannot be accessed outside the app. Apache Kafka is the core of the streaming service to ensure reliability, high availability, and scalability. All communications are transmitted using the Secure Sockets Layer (SSL) standard protocol, and data are encrypted at rest to ensure security. App access is controlled using a secure username-password combination.

## Discussion

### Principal Findings

Self-management of GDM is vital for controlling blood glucose levels and minimizing complications for both mother and baby [10,52]. In this paper, we described the design and development of GDM-DH, a culturally tailored GDM management app targeted for use among patients and health care providers in Nepal. Following the SCT framework [53], the GDM-DH app assists in self-management of GDM by enhancing the patients’ knowledge of and self-efficacy in adhering to blood glucose monitoring and recommended diet and physical activity regimens. With respect to the health care providers, the app’s web-based portal offers easy data visualization to track patient progress and treatment response, facilitating informed clinical decision-making at the point of care.

The growing literature highlights the importance of culturally tailoring health interventions, that is, adapting the intervention content and instructions according to the target users’ culture, diet, language, religion, customs, and beliefs [54-56]. Several studies [56-59], including systematic reviews [60], have found that culturally tailored programs and interventions are effective at improving disease knowledge, behavioral outcomes (eg, physical activity), access to care, and clinical outcomes, including glycated hemoglobin (HbA1C) levels in patients with diabetes. Our GDM-DH app development incorporated a user-centered design approach that actively involved end users and used ethnographic and human-computer interaction methodologies to better understand and meet their needs. the user-centered design approach is especially paramount to developing culturally tailored mobile interventions, ensuring app engagement, and promoting digital health equity in low-income countries, such as Nepal [54].

Although mobile technology has been widely applied and proven efficacious for self-management of diabetes outside of pregnancy [29,32,41,61], mobile app–based lifestyle interventions for GDM are just emerging, even in high-income countries [62-68]. To date, there are only a few published randomized controlled trials that have evaluated mobile app–based solutions for GDM management [63-68]. Two recent reviews [69,70] on app-based interventions for GDM concluded that most existing studies were of moderate quality and were underpowered to detect effects on perinatal outcomes but, overall, indicated improved glycemic control in the mobile intervention groups compared to standard care alone. However, most existing app-based interventions for GDM management focus on remote blood glucose monitoring, with manual feedback from health care providers [24,69,70], which can be resource intensive and burdensome for both providers and participants, thus limiting the potential for widespread dissemination and impact, particularly in low-resource settings, such as Nepal. Additionally, despite evidence showing that lifestyle and T2D interventions based on behavior change theory are more effective [71-73], we found only 2 studies [67,68] that incorporated relevant theories in their mobile intervention for GDM. Furthermore, only 2 studies [65,67] involved input from target users during app design and development, which is critical for ensuring the effectiveness and acceptability of evidence-based interventions [74].

### Strengths

Our GDM-DH app overcomes existing limitations and represents an advance over previous mobile interventions for GDM as it provides a comprehensive solution for GDM management without the need for additional work from health care professionals and incorporates user-centered principles and theory-based BCTs to meet the specific needs and technological literacy of our target users. To the best of our knowledge, the GDM-DH app is also the first to contain a social support component by including a social network feature. Although the educational content and custom food tracker (and visual aids for calorie/carbohydrate estimation) in our GDM-DH app were specifically designed for our target population of Nepalese women, they can be easily adapted and scaled to other contexts and populations by applying similar user-designed principles.

### Limitations

Several limitations of the GDM-DH app and its development process are worth mentioning. First, the number of participants in our usability study was limited. Additionally, during usability testing, we may have observed the best-case scenario for comfort and confidence in using the app, leading us to overestimate the true usability and technological proficiency. Second, we did not use a structured framework, such as the Delphi method [75], to organize and structure the discussions to guide our GDM-DH app and intervention development, which will make it difficult for others to replicate our study procedures. Nonetheless, we used many of the elements of the Delphi framework, including an iterative approach, the use of experts, and group-based responses. Similarly, although we did not use a structured framework to guide the modification and optimization of the GDM-DH app and intervention content, our prespecified criteria, including feasibility, scalability, and affordability, align with existing frameworks designed to optimize and evaluate an intervention prior to implementation (eg, multiphase optimization strategy, or MOST [76], and Acceptability, Practicability, Effectiveness, Affordability, Spill-Over Effects, and Equity, or APEASE [77], criteria). Due to resource limitations, we were unable to address all the needs and suggestions provided by our target users. For instance, the current version of the app has limited social support, but future versions could incorporate features such as a discussion forum to foster a stronger network and support system among users. The manual entry of blood glucose and blood pressure levels is also a limitation. Since the app was designed to address the cultural barriers and technological literacy of a specific population, generalizability is a potential issue, and adapting the app to other settings and contexts would require a similar level of user research and testing among the target populations. Large-scale cluster-randomized clinical trials at multiple urban and rural sites in Nepal are needed to establish the effectiveness and generalizability of the GDM-DH app to women with GDM across the country.

### Future Research

In the future, we plan to further optimize the GDM-DH app by including Bluetooth-enabled data entry and advanced smart phone functionalities, such as multimedia push notifications, and gamification features, which have been shown to increase retention and improve engagement with mHealth interventions [78-80]. Push notifications enable on-the-go delivery of intervention content, providing the necessary trigger and reinforcement when the specific intervention is most needed or is most convenient for the user. Gamification (application of game elements) features provide entertainment and intrinsic/extrinsic motivation (eg, point scores, badges, levels, a leaderboard) to promote sustained engagement with the app [81-85].

### Conclusion

The GDM-DH app targets specific needs identified by our target population in our pilot research and has unique features, including a social support feature, visual aids for carbohydrate estimation, and a comprehensive support system without imposing an additional provider burden. A proof-of-concept pilot clinical trial (NCT04198857) to study the feasibility, acceptability, and preliminary efficacy of the GDM-DH app is currently underway.

## Abbreviations

BCT: behavior change technique
GDM: gestational diabetes mellitus
GDM-DH: *Garbhakalin Diabetes athawa Madhumeha*—Dhulikhel Hospital
GWG: gestational weight gain
HIPAA: Health Insurance Portability and Accountability Act
KII: key informant interview
mHealth: mobile health
MVP: minimum viable product
PEN: Package of Essential Noncommunicable
SCT: social cognitive theory
T2D: type 2 diabetes

## Data Availability

The data sets generated and analyzed during this study are available from the corresponding author upon reasonable request.
